# Deletion of phosphatidylethanolamine methyltransferase promotes the spontaneous development of hepatic steatosis, inflammation, and fibrosis in young mice

**DOI:** 10.1042/CS20250454

**Published:** 2026-06-29

**Authors:** Sathish Kumar Perumal, Ramachandran Rajamanickam, Madan Kumar Arumugam, Srinivas Chava, Ramesh Bellamkonda, Sundararajan Mahalingam, Natalia A. Osna, Isis Trujillo-Gonzalez, Kurt W. Fisher, Karuna Rasineni, Kusum K. Kharbanda

**Affiliations:** 1 Research Service, Veterans Affairs Nebraska-Western Iowa Health Care System Omaha, Nebraska 68105, U.S.A.; 2 Department of Internal Medicine, University of Nebraska Medical Center, Omaha, Nebraska 68198, U.S.A.; 3 Center for Molecular and Nanomedical Sciences, Sathyabama Institute of Science and Technology, Chennai 600119, India; 4 Department of Biochemistry & Molecular Biology, University of Nebraska Medical Center, Omaha, Nebraska 68198, U.S.A.; 5 Department of Pharmacology and Experimental Neuroscience, University of Nebraska Medical Center, Omaha, Nebraska 68198, U.S.A.; 6 Department of Nutrition, Gillings School of Public Health, Nutrition Research Institute, University of North Carolina at Chapel Hill, Kannapolis, NC 28081, U.S.A.; 7 Department of Pathology, Microbiology and Immunology, University of Nebraska Medical Center, Omaha, Nebraska 68198, U.S.A.

**Keywords:** hepatic fibrosis, hepatic inflammation, hepatic steatosis, liver injury, phosphatidylcholine, Phosphatidylethanolamine methyltransferase

## Abstract

Phosphatidylethanolamine N-methyltransferase (PEMT) catalyzes the transfer of methyl groups to phosphatidylethanolamine to generate phosphatidylcholine (PC). PC produced *de novo* through this pathway is preferentially used for very-low-density lipoprotein assembly and is essential for its normal hepatic secretion as well as for bile acid detoxification. While human PEMT loss-of-function polymorphisms are linked to increased metabolic dysfunction-associated steatotic liver disease risk, the enzyme’s role as a primary driver of progressive liver disease remains underexplored. We utilized PEMT knockout (PEMT KO) mice on a standard chow diet to model this deficiency. Histopathological analysis showed that while 2-month-old PEMT KO mice were protected, 6-month-old KOs of both sexes spontaneously developed extensive micro- and macrovesicular steatosis, parenchymal inflammation, and granulomatous inclusions. This severe, age-dependent pathology was confirmed by elevated hepatic triglyceride levels, bile acids, impaired methylation potential, and a cascade of secondary injuries, including increased oxidative stress, impaired proteasomal function, and the induction of cellular senescence markers. This bile acid toxicity and oxidative stress activated the central innate immune sensor, the NLRP3 inflammasome, driving pronounced macrophage infiltration and chronic inflammation. Picrosirius red staining and protein analysis confirmed the progression to severe pericellular fibrosis. Our study establishes that PEMT deficiency is sufficient to initiate and drive the complete progression from steatosis to advanced liver fibrosis, identifying PEMT as a critical metabolic checkpoint and a potent therapeutic target for mitigating progressive liver disease.

## Introduction

Chronic liver disease (CLD) is a major global public health issue [1]. The causes of chronic liver injury include viruses, metabolic dysfunction, autoimmune diseases, and alcohol use disorder, all of which can lead to liver fibrosis [2,3]. Liver fibrosis can develop in patients with any type of CLD, including alcohol-associated liver disease (ALD), metabolic dysfunction-associated steatotic liver disease (MASLD), hepatitis C, hepatitis B, and autoimmune hepatitis [2]. Fibrosis, the most notable characteristic of CLD, involves both quantitative and qualitative alterations in the extracellular matrix, primarily the deposition of collagen. These changes can eventually lead to the complete disarray of liver architecture, resulting in cirrhosis and hepatocellular carcinoma development [1]. Historically, liver fibrosis was believed to be irreversible. However, extensive research has demonstrated that both fibrosis and cirrhosis can improve, especially when the root cause of liver injury is treated [4].

Phosphatidylethanolamine N-methyltransferase (PEMT), a transferase enzyme (EC 2.1.1.17) catalyzes the *de novo* synthesis of phosphatidylcholine (PC) in the liver [5–8]. This 22.3 kDa integral transmembrane protein is localized to the endoplasmic reticulum and mitochondria associated membranes of liver [9]. In mammals, PEMT catalyzes the three sequential transfers of methyl groups to phosphatidylethanolamine (PE) using S-adenosylmethionine (SAM) as the methyl donor to generate PC moieties [10–12], which are more diverse and composed of significantly longer chain, polyunsaturated fatty acids [8]. Further studies revealed that pharmacologic inhibition of PE methylation or deletion of PEMT impaired normal biogenesis and secretion of very-low-density lipoprotein (VLDL) by hepatocytes *in vitro* [13,14]. Conversely, stable expression of PEMT has been shown to enhance this secretion [14]. As the liver exports triglycerides and cholesterol only as constituents of VLDL particles, any impairment in either the synthesis or export of VLDL particles could lead to fat accumulation within the hepatocyte.

The PEMT-mediated catalysis is closely dependent on the availability of SAM, the methyl donor, and the ratio of SAM to S-adenosylhomocysteine (SAH) that provides a key index of the methylation potential [15]. Our laboratory has previously reported that alcohol-induced methylation defects (i.e. lower SAM:SAH ratio) impair PEMT activity to inhibit VLDL secretion, thereby contributing to hepatic steatosis development [16,17].

A recent study in aged mice (22–24-month-old) PEMT knockout (PEMT KO) fed a standard chow revealed no aging-related changes in the liver metabolome compared to the young (>2-months-old) KOs [18]. There were however increased levels of metabolites (adenosine monophosphate, histidine, inosine, acetate acid, propionate, glutathione) and decreased phosphorylcholine and choline levels in the young KOs compared to their age-matched wildtypes (WT) that persisted with aging of the KOs [18]. These studies did not particularly evaluate liver injury in depth nor looked at a relatively younger age of 6 months.

In humans, common *PEMT* genetic variants impair PC synthesis, and are associated with increased risk of developing MASLD [19,20]. Further, patients diagnosed with metabolic dysfunction-associated steatohepatitis (MASH) exhibit lower hepatic PEMT expression compared to those with simple steatosis that significantly correlated with platelet counts, which typically decline with advanced fibrosis [21]. Given that common genetic loss-of-function polymorphisms in the human *PEMT* gene predispose individuals to MASLD [19], we proposed that mice lacking PEMT would exhibit a progressive age-related liver damage at a relatively young age even when maintained on a normal diet.

## Materials and methods

### Animal handling and diet

PEMT KO mice were originally generated in Dr. Dennis Vance’s laboratory by targeted disruption of the *Pemt* gene through homologous recombination in embryonic stem cells, as described [22]. This strategy removed exons essential for PEMT enzymatic activity, resulting in complete loss of hepatic PEMT function. The line was backcrossed onto a C57BL/6 background and generously provided by Dr. René L. Jacobs (University of Alberta, Edmonton, Alberta, Canada).

Mice were housed and bred in the AAALAC-accredited Omaha Veterans Affairs Medical Center Veterinary Medical Unit under controlled environmental conditions (12-h light/12-h dark cycle, 20°C–25°C, 40%–60% relative humidity) with *ad libitum* access to standard chow and water. All procedures involving animals were approved by the Omaha Veterans Affairs Medical Center Institutional Animal Care and Use Committee (IACUC; IRBNet #1576244 and #1583585) and were conducted in accordance with institutional and federal guidelines.

For the present study, 2- and 6-months-old male and female C57BL/6 PEMT KO mice and their age- and sex-matched WT controls were euthanized under isoflurane anesthesia (5% to obtain anesthesia, 1%–2% to maintain anesthetized state), and blood was collected from the vena cava. The chest was opened, and death occurred from a combination of exsanguination and pneumothorax. These methods are consistent with the recommendations of the Panel on Euthanasia of the American Veterinary Association and were approved by the Omaha VA Animal Research Subcommittee. Organs were collected from each mouse. Serum was prepared by centrifuging whole blood in serum separator tubes at 13,000 × ***g*** for 5 min. Portions of each liver were immediately fixed in formalin for histologic analyses or processed for the preparation of a deproteinized extract using perchloric acid for SAM and SAH analyses, as detailed [23]. The remainder of each liver was freeze-clamped and stored at −70°C for subsequent biochemical assays.

### Hepatic histopathological analysis

Formalin-fixed liver tissues were processed, embedded in paraffin, sectioned, and stained with hematoxylin and eosin (H&E) for histopathological evaluation. A board-certified pathologist (K.W.F.) examined and graded the liver sections according to the Kleiner Criteria [24] that categorize the severity of hepatic steatosis on a scale from Grade 1 (mild) to Grade 3 (severe).

To assess fibrosis, deparaffinized sections were stained with picrosirius red staining (Cat# SSC1216-250, Cancer Diagnostics, Durham, NC, USA) for 60 min at room temperature. The slides were then incubated in 0.5% glacial acetic acid (Fisher chemicals) for 2 min and washed with water, followed by dehydration steps and mounted. For each independent experiment, all slides used for staining were processed at the same time to minimize variation in staining intensity. Digital images were acquired using a Keyence BZ-X810 microscope (Plano, TX, USA).

### Hepatic SAM, SAH, triglyceride, bile acids, and serum alanine aminotransferase levels

We subjected liver perchloric acid extracts to high-performance liquid chromatography (HPLC) to quantify SAM and SAH levels, as detailed [23,25]. The triglyceride levels in liver lipid extracts [26] were quantified using the diagnostics kit (Cat# TR22421, Thermo Fisher Scientific, Middleton, VA, USA) using the manufacturer’s instructions, as detailed previously [23].

Since maintaining hepatic PC is critical for biliary secretion [27], we also measured hepatic bile acids using bile acid assay kit (Cat# STA-631, Cell Biolabs Inc., San Diego, CA, USA).

Serum levels of alanine aminotransferase (ALT) were measured at the clinical laboratory at VA Nebraska Western Iowa Health Care System.

### Hepatic betaine and betaine–homocysteine methyltransferase activity

The betaine–homocysteine methyltransferase (BHMT) enzyme activity in the liver samples was measured following the method of Ericson and Harper and expressed as mg methionine formed [28]. Liver betaine levels were measured by liquid chromatography–electrospray ionization-isotope dilution mass spectrometry as previously described [29].

### Reactive oxygen species and thiobarbituric acid-reactive substances

Liver reactive oxygen species (ROS) were measured using 2′7′-dichlorodihydrofluorescein diacetate as detailed [30]. Formation of the oxidized fluorescent derivative, dichlorofluorescein, was monitored at 485 nm (excitation) and 530 nm (emission). Data are expressed as fluorescence units normalized for protein concentration, which was measured by the Bradford dye-binding assay [31]. We measured hepatic lipid peroxidation by thiobarbituric acid-reactive substances (TBARS) assay, as detailed [32] using purified malondialdehyde (MDA) as the standard.

### Proteasome activity

Liver homogenates were used to assay the trypsin- and chymotrypsin-like activity of the proteasome using appropriate fluorometric substrates and buffers according to established procedure as previously described [33–35]. Data are expressed as fluorescence units per hour normalized for protein concentration [31].

### Lysosomal hydrolases

We determined the catalytic activities of three major hepatic lysosomal hydrolases, cathepsin B, cathepsin L and the lysosomal acid lipase (LAL), in the homogenates using the appropriate fluorometric 4-methylcoumaryl-amide substrates and buffers according to established procedures [36,37].

### Gene expression

RNA was isolated with a PureLink™ RNA minikit (Cat# 12183018A, Invitrogen, Waltham, MA, USA) following the manufacturer’s instructions. RNA was quantified spectrophotometry (NanoDrop Technologies, Wilmington, DE, USA), and 200 ng of RNA was reverse transcribed to cDNA using a high-capacity reverse-transcription kit (Cat# 4368813, Applied Biosystems, Waltham, MA, USA). For quantitative PCR, synthesized cDNA was amplified by real-time PCR (7500 Fast Real-Time PCR system, Applied Biosystems, Waltham, MA, USA) using iTaq Universal SYBR Green Supermix (Cat# A25742, Applied Biosystems, Waltham, MA, USA). The relative quantity of each RNA transcript was calculated by its threshold cycle after subtracting that of the reference cDNA (β-actin). Data are expressed as the relative quantity of each transcript. Detailed information on the primers is presented in Table 1.

**Table 1:** Primers sequences used for determining mRNA expression

Gene name	Sequence (5′-3′)	Accession #
*Cyp7a1*	Forward: GGGCAGGCTTGGGAATTTTG Reverse: AACGCTCAGCAGTCGTTACA	NM_007824.3
*Cyp8b1*	Forward: TTGCAAATGCTGCCTCAACC Reverse: TAACAGTCGCACACATGGCT	NM_010012.3
*β-actin*	Forward: ATGCCCTGAGGCTCTTTTCC Reverse: CAGCTCAGTAACAGTCCGCC	NM_007393.5

### Immunofluorescence staining

Immunofluorescence staining was performed as previously described [38]. Briefly, paraffin-embedded liver sections were deparaffinized in xylene and rehydrated through a graded ethanol series. Following deparaffinization, slides were subjected to antigen retrieval using 10 mmol/l sodium citrate buffer (pH 6.0) at 95°C for 15 min. Nonspecific binding was blocked by incubation with Super Block (Cat# AA999, ScyTek Laboratories, Logan, UT, USA) for 1 h at room temperature. Sections were then incubated overnight at 4°C with primary antibodies against F4/80, MDA, or 4-hydroxynonenal (HNE). After thorough washing with PBS, sections were incubated with ImmPRESS^®^-AP Horse Anti-Rabbit IgG Polymer Reagent, Alkaline Phosphatase (Cat# MP-5401, Vector Laboratories Inc., Newark, CA, USA) according to the manufacturer’s instructions followed by exposure to Vector Red Substrate Kit, Alkaline Phosphatase (Cat# SK-5100, Vector Laboratories Inc., Newark, CA, USA) for signal development. Nuclei were counterstained by incubating the sections with 4′,6-diamidino-2-phenylindole (DAPI; 1 μg/ml) for 1 min and the slides were imaged using a Keyence BZ-X810 microscope. Antibody details are provided in Table 2.

**Table 2:** List of primary and secondary antibodies used in experiments

Primary antibody	Host	Cat. no.	Manufacturer
β-actin	Mouse	66009-1-Ig	Protein Tech
MDA	Mouse	MA5-27560	Invitrogen
4-Hydroxynonenal (4-HNE)	Mouse	MA5-27570	Invitrogen
p53/TRP53	Mouse	MA5-12453	Invitrogen
p21/CDKN1A	Rabbit	14-6715-81	Invitrogen
Nucleotide-binding domain, leucine-rich repeat, and pyrin domain-containing protein 3 (NLRP3)	Mouse	68102-1-Ig	Protein Tech
Interleukin-1β (IL-1β)	Mouse	1242S	Cell signaling technology (CST)
Cluster of differentiation 68 (CD68)	Rabbit	Ab125212	Abcam
F4/80	Rabbit	70076	CST
Transforming growth factor β (TGF-β)	Mouse	sc-130348	Santa Cruz
Tissue inhibitors of metalloproteinases 2 (TIMP2)	Rabbit	ab180630	Abcam
Smooth muscle actin-α (α-SMA)	Mouse	A2547	Sigma
Collagen II	Mouse	NB600-488	Novus

Secondary antibody	Host	Cat. No.	Manufacturer
Peroxidase AffiniPure Goat Anti-Rabbit IgG	Goat	111-035-144	Jackson ImmunoResearch laboratories
Peroxidase AffiniPure Goat Anti-Mouse IgG	Goat	115-035-166	Jackson ImmunoResearch laboratories

### Western blotting

Liver homogenates were subjected to Western blot analysis using primary antibodies directed against MDA, HNE, cyclin-dependent kinase Inhibitor (p21), tumor protein 53 (p53), CD68, NLRP3, IL-1β, TGF-β, TIMP2, or β-actin as we previously described [20,23,39]. The same β-actin blot was used as a loading control for multiple targets (MDA, HNE, p53, p21, CD68, IL-1β, and NLRP3), as all corresponding Western blots were performed using the same lysate and processed concurrently. Table 2 provides detailed information on all antibodies used in the present study. Immunoreactive proteins were visualized using an enhanced chemiluminescence substrate (Cat# 170-15060; Bio-Rad Laboratories, Hercules, CA, USA) on a ChemiDoc MP imaging system (Bio-Rad Laboratories), and band intensities were quantified using ImageJ software (version 1.54g).

### Statistical analysis

Data were analyzed by ANOVA followed by Tukey post-hoc test for comparisons among groups and results considered statistically different at a probability *P*-value ≤0.05.

## Results

### PEMT deficiency promotes hepatic steatosis, liver injury, and altered bile acid metabolism in 6-month-old mice

**Figure 1:**
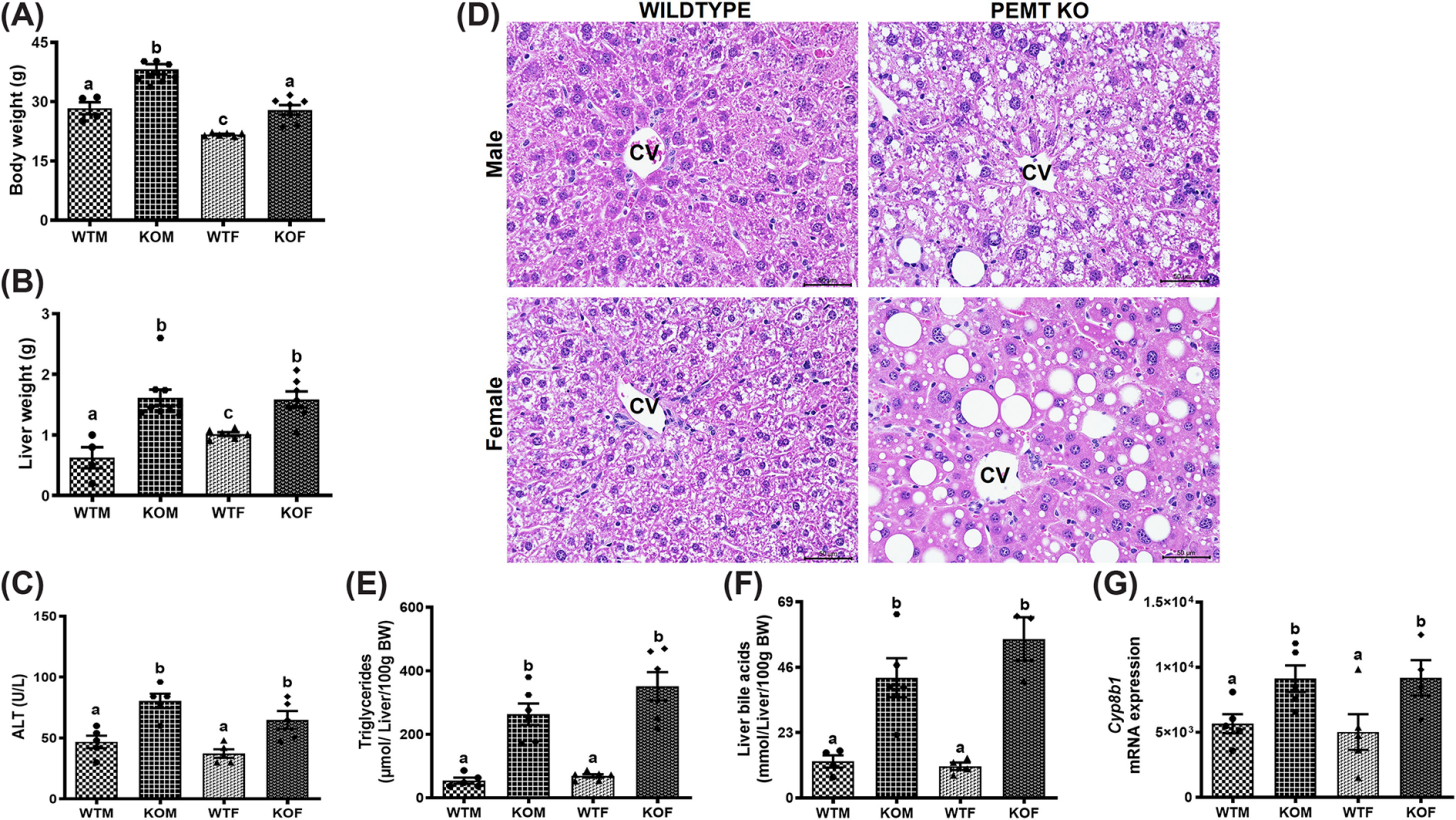
PEMT deficiency promotes spontaneous development of hepatic steatosis, liver injury and bile acid accumulation (**A**) Body weight, (**B**) liver weight, (**C**) serum ALT levels, (**D**) representative images of H&E-stained liver sections (scale bar: 50 μm), (**E**) hepatic triglycerides, (**F**) bile acids, and (**G**) mRNA expression encoding for cytochrome P450 family 8 subfamily B member 1 (CYP8B1) in 6-month-old male and female PEMT KO (KOM and KOF, respectively) compared with their respective age- and sex-matched wildtype (WTM and WTF) mice. Data are presented as the mean ± SEM (*n* = 4–6); values not sharing a common letter significantly differ from each other at *P*≤0.05.

Body weight, liver weight, liver histology, and hepatic triglyceride levels were comparable between 2-month-old male and female PEMT KO mice and their respective sex- and age-matched WT controls (Supplementary Figure S1). Therefore, all subsequent analyses were conducted in 6-month-old mice. At this age, PEMT KO mice of both sexes displayed significantly higher body, liver weights and serum AST levels compared with their corresponding WT groups (Figure 1A–C). Histopathological examination showed normal hepatic architecture in WT mice, whereas PEMT KO mice exhibited pronounced vacuolated regions consistent with lipid accumulation in hepatocytes surrounding the central vein (Figure 1D). Further biochemical quantification corroborated our histological results as we observed elevated hepatic triglyceride levels in livers of 6-month-old PEMT KO mice when compared to their respective age- and sex-matched WT mice (Figure 1E). Notably, although the representative images of male and female PEMT KO mice appear to differ in lipid droplet size or distribution, quantitative analysis of hepatic triglyceride levels showed no statistically significant differences between the two groups, although female KOs exhibited slightly higher numerical values (Figure 1D). Steatosis scoring of H&E-stained liver tissues following Kleiner Criteria [24] did not reveal any difference in the grading, or percentage of micro- and macrosteatosis between the male and female KOs (Table 3).

**Table 3:** Hepatic steatosis grading

	Male		Female	
	WT	PEMT KO	WT	PEMT KO
**Steatosis score**	0^a^	1.13 ± 0.18^b^	0^a^	1.43 ± 0.20^b^
**Microsteatosis (%)**	0^a^	12.31 ± 0.03^b^	0^a^	10.00 ± 0.03^b^
**Macrosteatosis (%)**	0^a^	20.50 ± 0.06^b^	0^a^	21.43 ± 0.03^b^

Values are mean ± SEM (*n* = 6); values not sharing a common letter significantly differ from each other at *P*≤0.05.

In addition, PEMT KO mice of both sexes exhibited increased hepatic bile acid levels compared to their respective WTs (Figure 1F). While the mRNA expression encoding for the first and rate limiting step in bile acid synthesis, cholesterol 7-alpha-monooxygenase (CYP7A1), remained unchanged between PEMT KO and sex- and age-matched WT mice (data not shown), significantly elevated mRNA expression encoding for sterol 12-α-hydroxylase also known as CYP8B1, an enzyme that catalyzes a key step in the conversion of cholesterol to cholic acid, one of the primary bile acids (Figure 1G).

### PEMT deficiency alters parameters related to methionine metabolic pathway

**Figure 2:**
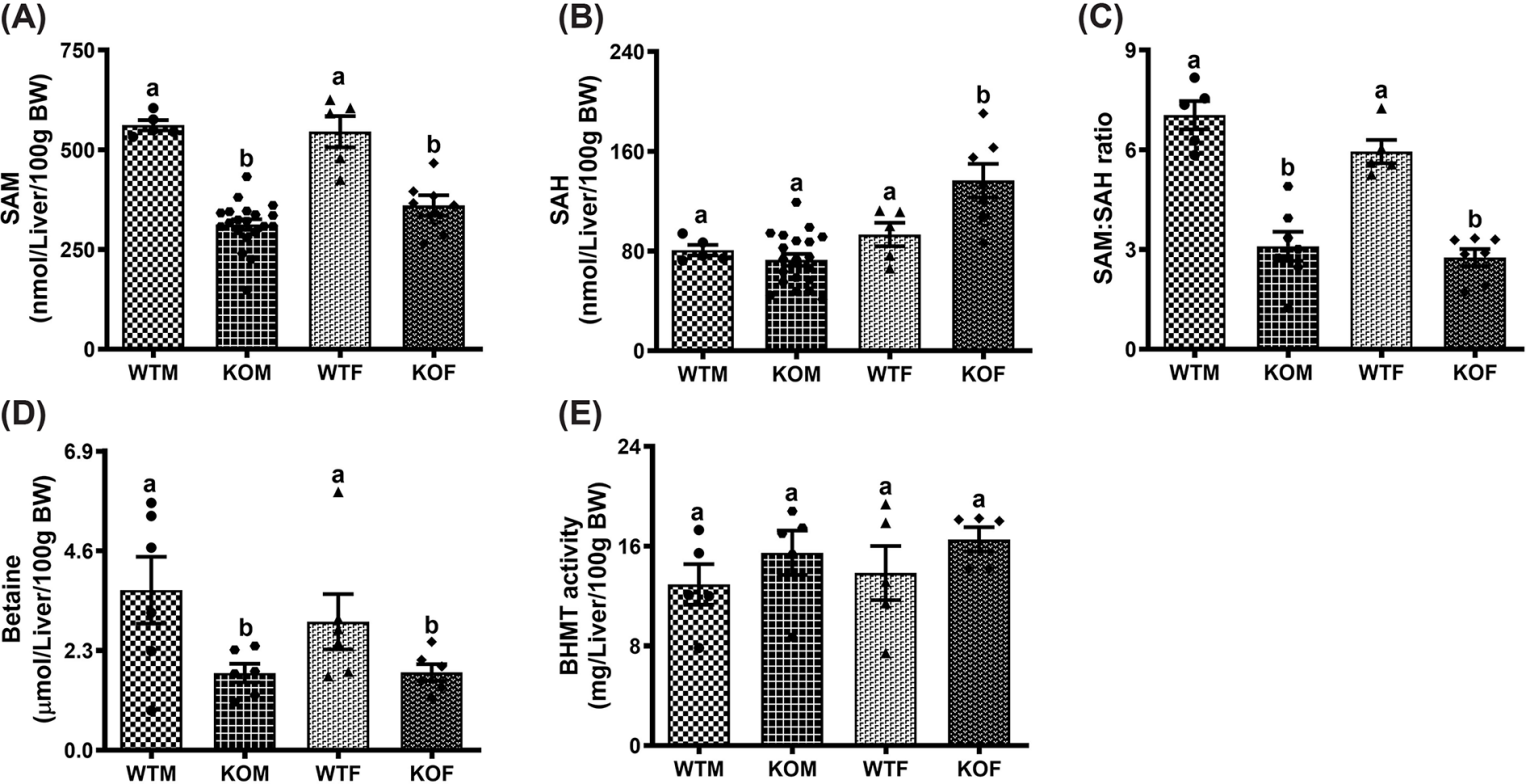
PEMT deficiency impairs hepatic methylation potential (SAM:SAH ratio) and betaine levels without affecting BHMT activity Hepatic (**A**), SAM, (**B**) SAH, (**C**) SAM:SAH ratio, (**D**) betaine levels, and (**E**) BHMT enzyme activity (expressed as methionine formed) in male and female wildtype (WTM and WTF) and PEMT KO (KOM and KOF) mice. Data are presented as means ± SEM (*n* = 5–8); values not sharing a common letter are statistically different, *P*≤0.05.

HPLC analysis of liver tissues revealed significantly lower hepatic SAM levels and SAM:SAH ratios in PEMT KO mice compared with their matched WT controls (Figure 2A,C). SAH levels were unchanged in males (Figure 2B), whereas female KOs exhibited moderately but significantly elevated SAH levels relative to the other groups. There was also a decrease in hepatic betaine levels in PEMT KO of both sexes compared to their counterpart WTs (Figure 2D) while there was minimal change in the activity of the enzyme, BHMT, that utilizes betaine (Figure 2E) to regenerate SAM.

### PEMT deficiency modulates proteasome and lysosomal hydrolases activities

**Figure 3:**
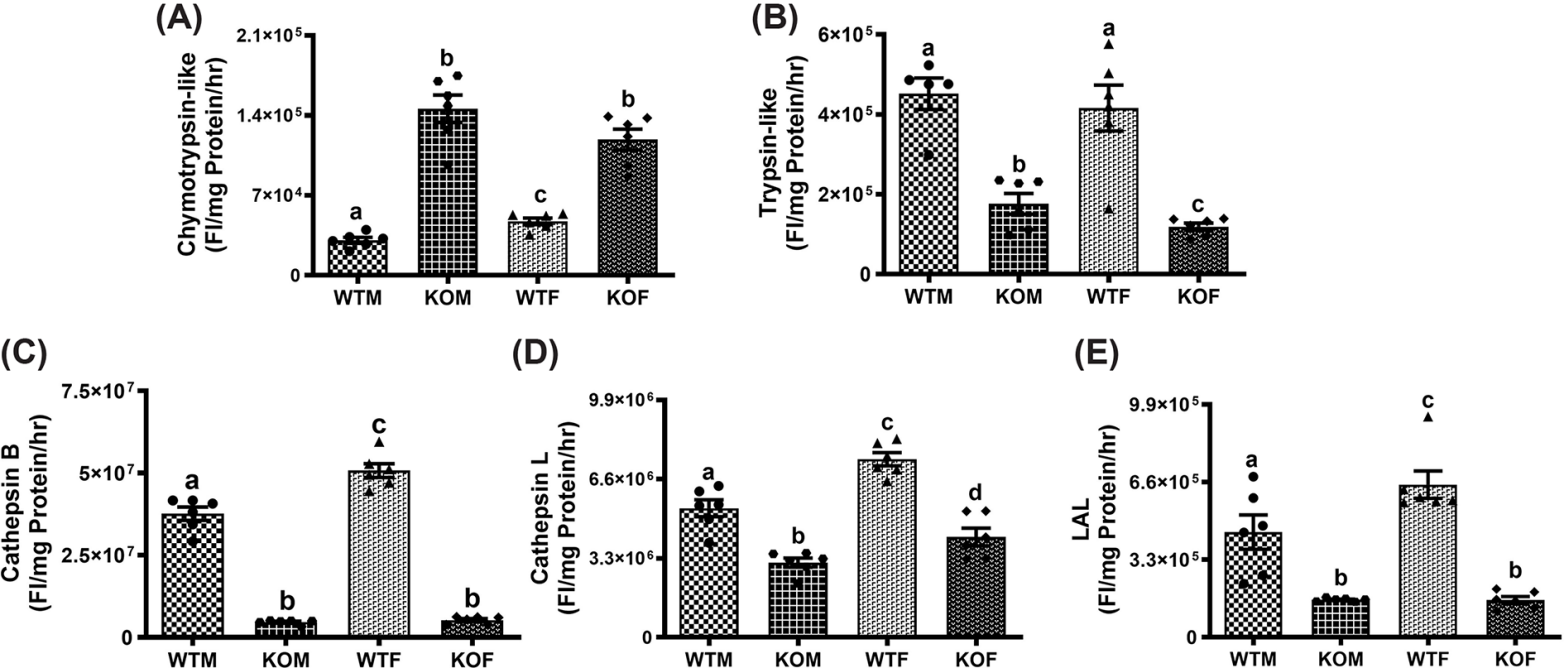
PEMT deficiency alters proteasomal and lysosomal hydrolases activities (**A**) Proteasomal chymotrypsin-like, (**B**) proteasomal trypsin-like, (**C**) lysosomal cathepsin (**B,D**) cathepsin L, and (**E**) LAL activities in the livers of 6-month-old male and female wildtype (WTM and WTF) and PEMT KO (KOM and KOF) mice. Data are presented as the mean ± SEM (*n* = 5–8); values not sharing a common letter significantly differ from each other at *P*≤0.05.

Alteration of lipid metabolism and cellular stress in PEMT KO mice increased the proteasomal degradation activities as evident from chymotrypsin-like proteosome activities in these mice compared to WT mice (Figure 3A). In contrast, the trypsin-like proteasome (Figure 3B) as well as lysosomal hydrolase activities that play important roles in cellular homeostasis, tissue remodeling and disease progression showed a significant decrease in the KOs. We observed a significant reduction in hepatic cathepsin B and cathepsin L activities in PEMT KOs ice compared to age-matched WT mice (Figure 3C,D). LAL activity is a crucial element, whose impairment leads to the accumulation of triglycerides in the liver [40]. PEMT deficiency resulted in a significant reduction in LAL activity in both male and female mice. Specifically, LAL activity was reduced by approximately 3.7-fold in PEMT KO males and ∼4.9-fold in PEMT KO females relative to their WT controls (Figure 3E).

### PEMT deficiency increases ROS production and lipid peroxidation

**Figure 4:**
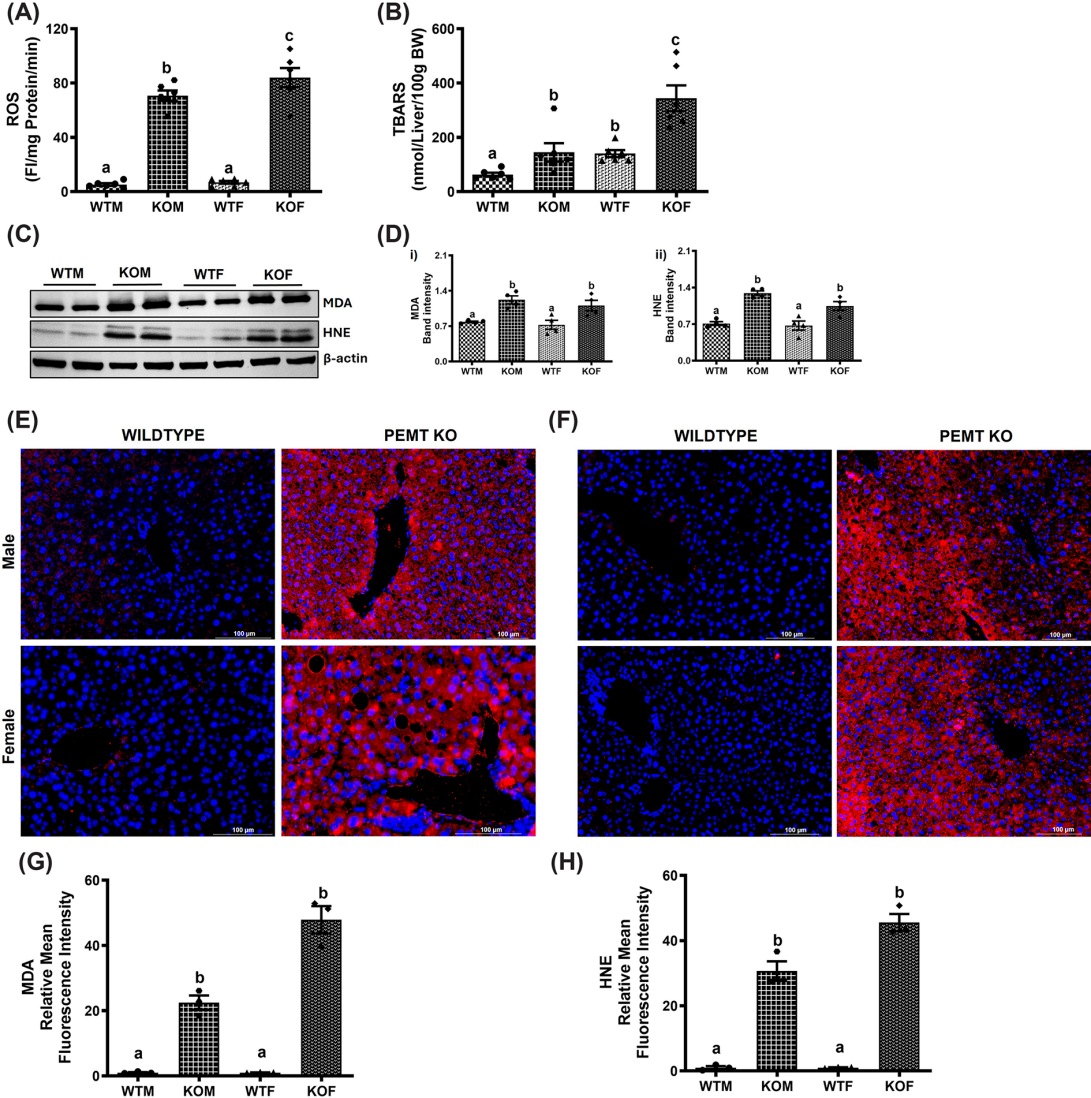
PEMT deficiency increases oxidative stress Levels of (**A**) ROS, (**B**) TBARS in the liver of 6-month-old male and female wildtype (WTM and WTF) and PEMT KO (KOM and KOF) mice. (**C**) Representative Western blot of liver lysates showing the formation of proteins adducts with lipid peroxidation products, MDA or 4-HNE and (**D**) with band intensities quantified using ImageJ and normalized to the β-actin loading control. (**E,F**) Representative fluorescent images (scale bar: 100 μm) of liver sections immunostained for MDA or HNE (red) with DAPI nuclear counterstain (blue). (**G,H**) Quantification of fluorescent intensity showing levels of MDA- and HNE-protein adducts in male and female PEMT KO mice relative to sex-matched WT. Data are presented as the mean ± SEM (*n* = 4–6); values not sharing a common letter significantly differ from each other at *P*≤0.05.

PEMT deletion contributes to disrupted lipid metabolism and increased cellular oxidative stress. We observed significant elevations in both ROS and TBARS levels in PEMT KO mice compared with WT mice (Figure 4A,B). In addition, both Western blot and immunofluorescence analyses revealed marked increases in MDA- and HNE-modified protein adducts in PEMT KO mice of both sexes compared with WT counterparts (Figure 4C–H).

### PEMT deficiency promotes hepatic senescence and inflammation

**Figure 5:**
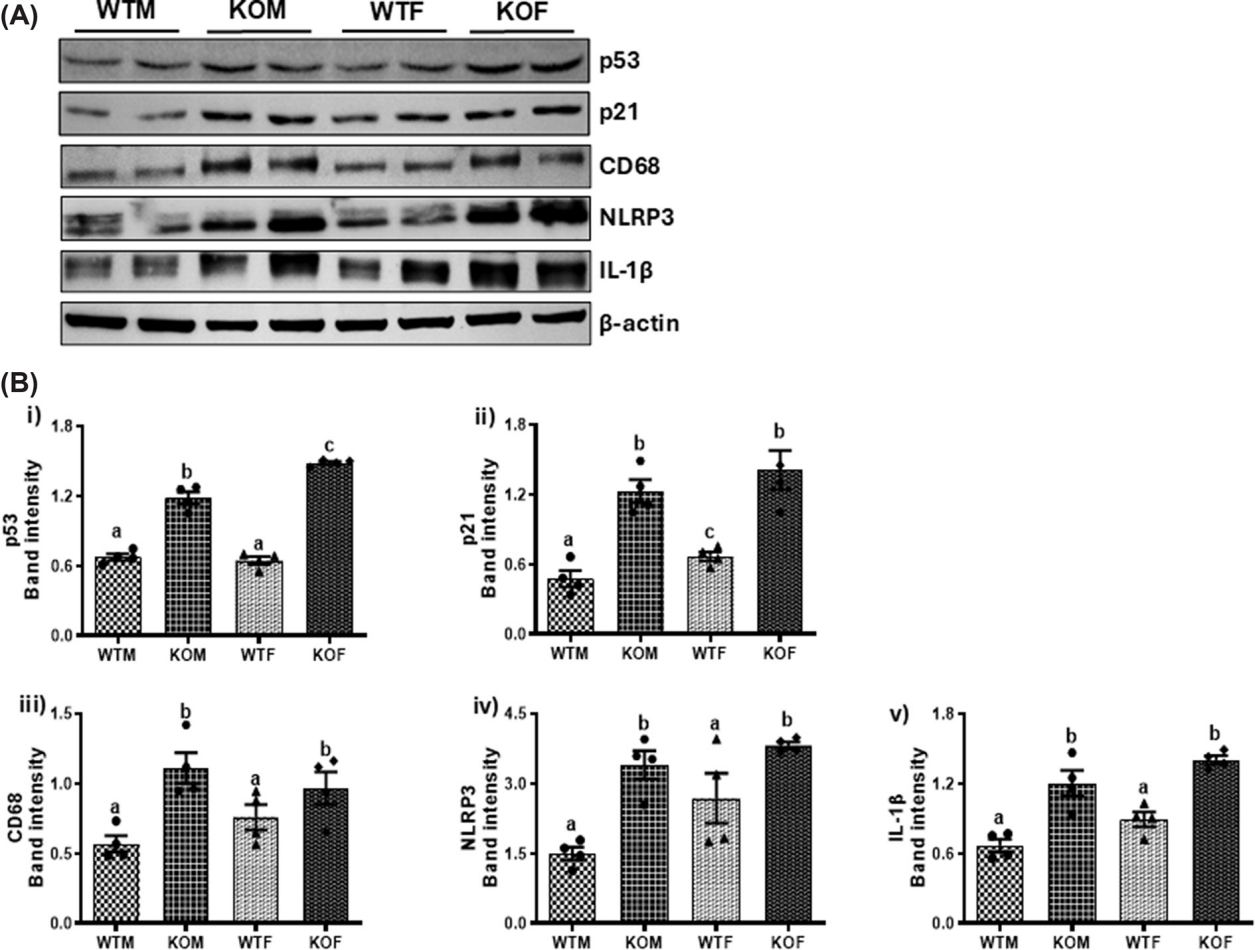
PEMT deficiency promotes hepatic senescence and inflammation (**A**) Western blot showing increases in cyclin-dependent kinase inhibitor (p21), tumor protein 53 (p53), CD68, NLRP3, and IL-1β in representative liver lysates of 6-month-old wildtype (WTM and WTF) and PEMT KO (KOM and KOF) mice and (**B**) with band intensities quantified using ImageJ and normalized to the β-actin loading control. Data are presented as the mean ± SEM (*n* = 4); values not sharing a common letter significantly differ from each other at *P*≤0.05.

**Figure 6:**
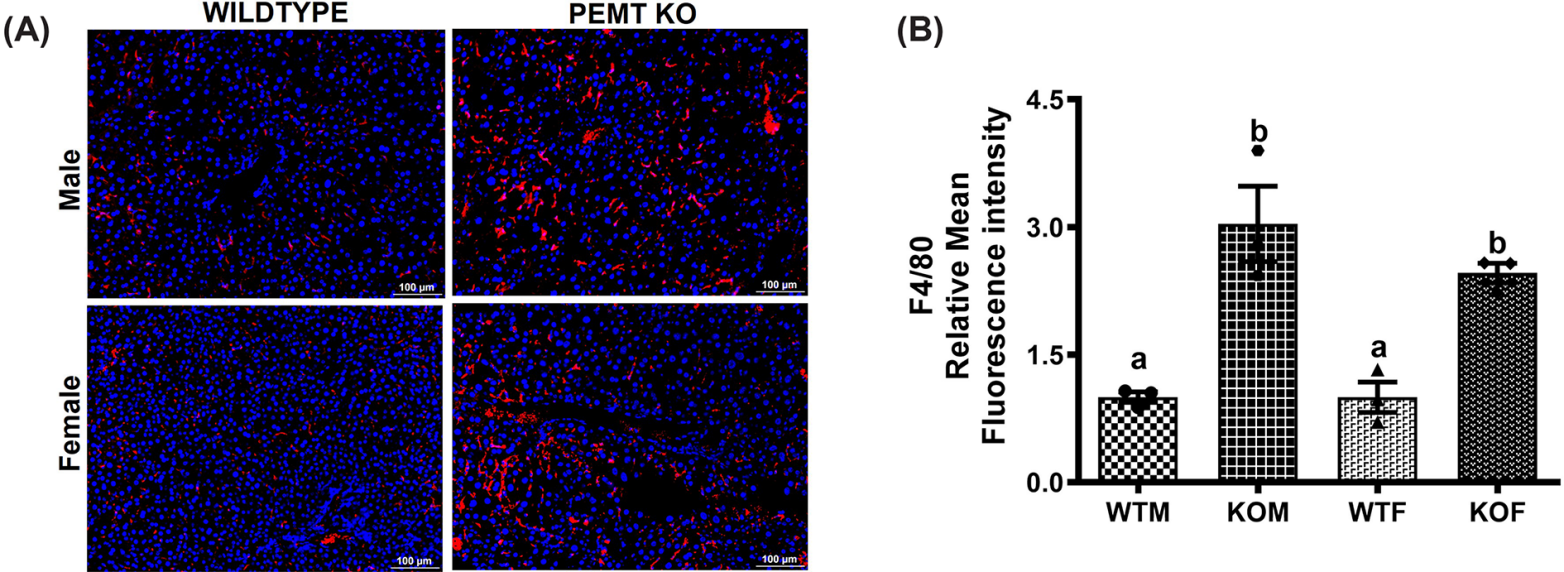
PEMT deficiency activates Kupffer cells (**A**) Representative fluorescent images (scale: 100 μm) of liver sections from male and female WT (WTM and WTF) and PEMT KO (KOM and KOF) mice immunostained with F4/80 (red) and counterstained with DAPI (blue). (**B**) Quantification of F4/80 fluorescent intensity relative to WT males. Values represent means ± SEM from four randomly selected fields per section, with data pooled from individual animals (*n* = 4); values not sharing a common letter significantly differ from each other at *P*≤0.05.

Deletion of PEMT not only disrupted hepatic lipid metabolism but also contributed to enhanced senescence and inflammation as evident from increases in markers of senescence (p53 and p21) and inflammation (CD68, NLRP3, IL-1β) in livers of PEMT KO mice when compared to age- and sex-matched WT mice (Figure 5A,B). Further, we found increased F4/80 staining in livers of PEMT KO mice compared to WT mice (Figure 6A,B).

### PEMT deficiency accelerates the development of liver fibrosis

**Figure 7:**
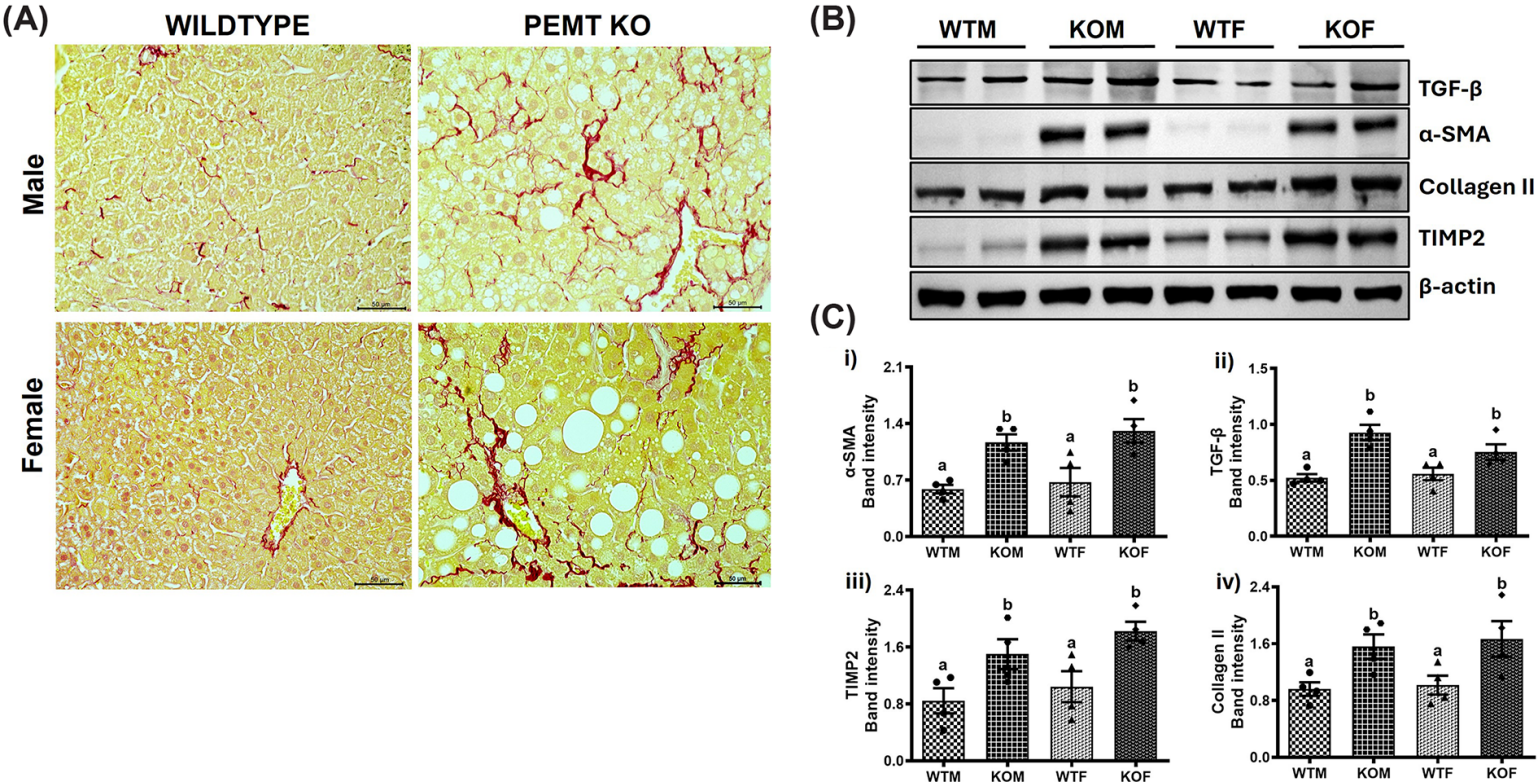
PEMT deficiency accelerates the development of liver fibrosis (**A**) Picrosirius red staining of representative liver sections of 6-month-old male and female wildtype (WTM and WTF) and PEMT KO (KOM and KOF) showing collagen bundles (scale: 50 μm). (**B**) Western blots showing increases in TGF-β1, α-SMA, collagen II, TIMP2, β-actin in representative liver lysates and (**C**) with band intensities quantified using ImageJ and normalized to the β-actin loading control. Values are mean ± SEM (*n* = 4); values not sharing a common letter significantly differ from each other at *P*≤0.05.

Picrosirius red staining revealed significantly increased hepatic fibrosis in PEMT KO mice compared with age-matched WT controls. Liver sections from PEMT KO mice exhibited a marked accumulation of collagen bundles relative to WT mice (Figure 7A). These histological findings were further supported by elevated protein expression levels of fibrotic markers, including TGF-β, α-SMA, collagen II, and TIMP2, in PEMT KO mice compared with age- and sex-matched WT mice (Figure 7B,C).

## Discussion

PEMT is a key enzyme in one-carbon metabolism that catalyzes the conversion of PE to PC using SAM as a methyl donor. Previous studies have demonstrated that reduced hepatic SAM levels or PEMT deficiency decreases PE methylation-mediated PC synthesis, thereby impairing VLDL secretion and promoting lipid accumulation in hepatocytes [14,17,23,41,42]. The liver primarily stores lipids as triglycerides, an inert and noncytotoxic form of lipid [43]. However, excessive lipid accumulation in hepatocytes is a major driver of oxidative stress, which can accelerate the progression of inflammation and fibrosis [44].

Our findings demonstrate that PEMT KO mice, irrespective of sex, develop spontaneous hepatic lipid accumulation characterized by increased lipid droplet number and elevated triglyceride levels compared with age- and sex-matched WT mice. While no significant differences in hepatic triglyceride levels were observed between 2-month-old PEMT KO and WT mice, triglyceride levels increased approximately 3–4-fold in PEMT KO mice by 6 months of age. Impaired hepatic triglyceride export via VLDL, resulting from deficient PEMT-mediated PC synthesis, appears to be the primary mechanism underlying intracellular lipid accumulation, spontaneous hepatic steatosis, and progressive liver injury. Consistent with this interpretation, hepatocellular damage was reflected by elevated ALT levels in PEMT KO mice compared with WT controls.

In addition to steatosis, PEMT KO mice displayed increased hepatic bile acid levels, suggesting either enhanced bile acid synthesis or impaired biliary secretion [45,46]. Because intracellular bile acid accumulation can activate immune cells and promote inflammation and fibrosis [27,47], the elevated bile acids likely contribute to the heightened inflammatory and fibrotic responses observed in the KO mice. This increase in hepatic bile acids is most consistent with impaired bile acid export, as PC synthesized through the PEMT pathway is essential for the function of the bile salt export machinery. PC is co-secreted with bile acids to form mixed micelles that neutralize their detergent properties. Biliary PC constitutes ∼40% of the organic fraction of bile and is critical for protecting hepatocytes and the biliary epithelium from bile acid-induced cytotoxicity. By converting bile acids from monomers and simple micelles into less-toxic mixed micelles, PC preserves membrane integrity and intestinal mucosal protection [45,48]. Thus, reduced PC availability in PEMT-deficient livers likely contributes to bile acid retention and the progression of liver injury. To further define the mechanisms underlying bile acid accumulation, we examined the expression of enzymes involved in bile acid synthesis. Although the rate-limiting enzyme CYP7A1 was unchanged between KO and sex-matched WT mice, CYP8B1 expression was significantly increased in PEMT-KO animals. Elevated CYP8B1 favors the synthesis of cholic acid, producing a more hydrophobic and detergent-rich bile acid pool that can exacerbate hepatocyte stress and bile acid toxicity. Together, these findings suggest that altered bile acid composition, specifically increased cholic acid synthesis, combined with impaired PC-dependent bile acid export drives the progressive liver injury associated with PEMT deficiency, rather than impaired secretion alone.

Furthermore, the development of steatotic liver disease in 6-month-old PEMT KO mice was associated with a significant reduction in the hepatic SAM:SAH ratio compared with age-matched WT mice. This finding was unexpected, given that PEMT is a major consumer of SAM [49]. We anticipated that PEMT deficiency would result in increased hepatic SAM levels and, consequently, an elevated SAM:SAH ratio. Instead, we observed the opposite effect, with a marked reduction in the SAM:SAH ratio in PEMT KO mice.

Previous studies from our laboratory have demonstrated that a reduced hepatocellular SAM:SAH ratio, with consequent impairment of multiple methylation reactions, represents a central pathophysiological mechanism underlying ALD [50,51]. A similar disruption in methylation potential is evident in PEMT KO mice. The decreased SAM:SAH ratio in these animals appears to primarily reflect impaired SAM regeneration due to reduced hepatic betaine availability. Betaine serves as the methyl donor for BHMT-mediated remethylation of homocysteine to sustain hepatic SAM levels; thus, its depletion severely limits methylation capacity. This mechanism closely parallels observations in ALD, where reduced betaine levels contribute to defects in hepatic methylation reactions [50–52]. In addition to diminished BHMT-mediated remethylation, secondary metabolic disturbances in PEMT-deficient livers may further exacerbate the decline in methylation potential. Increased oxidative stress is known to impair methionine synthase activity, the alternative remethylation pathway in one-carbon metabolism, thereby further limiting SAM regeneration and promoting SAH accumulation [53–55]. Collectively, these defects leading to a sustained reduction in the SAM:SAH ratio are likely key contributors to the progression of liver injury associated with PEMT deficiency.

Our previous studies have indicated an association between the reduction in the methylation potential and altered cellular degradation mechanisms. While proteasomal inhibition can result from a reduction in SAM:SAH ratio [34,56,57], in the present study we observed that while there was a decrease in the trypsin-like proteasomal activity, there was an increase in the chymotrypsin-like activity in the PEMT KOs compared to WT. This selective increase in the chymotrypsin-like activity of the proteasome is likely a compensatory response to the oxidative stress as reported [35] and an attempt to manage the growing load of irreversibly damaged cellular components, such as MDA- and HNE-protein adducts. Cathepsins are the most abundant lysosomal proteases that are mainly found in the acidic endo/lysosomal compartments where they play a vital role in intracellular protein degradation, energy metabolism, immune responses and cell survival [58]. We observed decreased activities of cathepsins B and L as well as LAL in PEMT KO mice when compared to WT mice. The loss of cathepsin and LAL activities are related to reduction in SAM:SAH ratio as treatment with betaine that normalizes SAM:SAH ratio also reverses the inhibition as recently reported [40]. Inhibition of the lysosomal enzyme activities could contribute to increased triglyceride accumulation in the KO mice via a reduction in LAL activity while a deficiency in cathepsin can cause toxic buildup resulting in cell stress, promote inflammation, cell death [59], and cellular senescence [60] as seen in the PEMT KOs.

The accumulation of lipids affects cellular function through different mechanisms including oxidative and endoplasmic reticulum stress, mitochondrial dysfunction, and induction of apoptosis [44]. In the present study, PEMT deletion contributed to hepatic fat accumulation and increased cellular oxidative stress as evident by increased TBARS, and levels of protein adducts with lipid peroxidation byproduct, MDA and HNE. These Western blot findings were further corroborated by immunofluorescence analysis, providing evidence for enhanced lipid peroxidation at the cellular level in PEMT-deficient livers. Binding of highly reactive lipid peroxidation byproducts to proteins and DNA disrupts the normal function of these macromolecules, leading to cellular dysfunction and, ultimately, cell death. Moreover, the formation of HNE- and MDA-protein adducts can elicit immune responses and contribute to disease pathogenesis [61,62].

Oxidative stress and chronic inflammation are well-established drivers of progressive liver injury, and substantial experimental and clinical evidence demonstrates that oxidative stress initiates inflammatory signaling and promotes its transition to fibrosis [63,64]. In the present study, the marked lipid accumulation in the livers of 6-month-old PEMT KO mice was accompanied by increased oxidative stress and a robust inflammatory response. This was evidenced by activation of Kupffer cells, enhanced macrophage infiltration, and elevated protein levels of key inflammatory mediators, including NLRP3 and IL-1β, compared with WT controls. We also observed a significant increase in the expression of the cell-cycle arrest markers p21 and p53 in PEMT-KO mice, consistent with hepatocellular stress and injury. Mechanistically, ROS and lysosomal damage are known activators of the NLRP3 inflammasome [65], which in turn drives the maturation and release of pro-inflammatory cytokines such as IL-1β [66]. In the present study, in line with increased levels of lipid peroxides and NLRP3 component, we have observed increased protein levels of IL-1β in PEMT KO mice. This cytokine perpetuates the inflammatory cycle by initiating cell death and recruiting peripheral immune cells. This dramatic increase in Kupffer cell activation, massive infiltration of CD68 positive macrophages coupled with enhanced NLRP3 signaling and cytokine release, collectively confirms that PEMT deficiency spontaneously drives the liver into a severe, self-sustaining inflammatory state, establishing the pathological bridge linking steatosis to the inevitable progression toward fibrosis.

Consistent with observed inflammation, picrosirius red staining revealed significant collagen bundles in the liver sections of PEMT KO mice of both sexes compared to their WT counterparts demonstrating hepatic fibrosis. This was further validated by the increased protein expression of fibrotic markers, including TGF-β, α-SMA, collagen II, and TIMP2. These findings confirm that the absence of PEMT promotes progressive liver fibrosis spontaneously in relatively young mice on a healthy chow diet without any external second insults such as alcohol, high-fat, or viral infection.

To summarize, we show that the absence of PEMT disrupts the SAM:SAH ratio in the liver that is accompanied by the spontaneous development of hepatic steatosis, inflammation, fibrosis at a relatively young age of 6 months compared to control mice. These effects arise from the loss of PEMT-mediated PC synthesis. We did not observe any significant differences in metabolic dysfunction between the female and male PEMT KO mice, except for higher levels of SAH, ROS, and TBARS in the females. These elevations may reflect sex hormone-dependent differences that increase female susceptibility to PEMT deficiency. Such sex dependent effects are biologically plausible, as estrogen normally enhances PEMT expression, influences SAM:SAH homeostasis, and modulates antioxidant defenses. In the absence of PEMT, these estrogen-regulated pathways may become dysregulated, contributing to the greater accumulation of methylation intermediates and oxidative stress observed in females.

Based on the findings in the present study, we propose that loss-of-function polymorphisms in PEMT may have similar consequences in humans as they age despite being on a balanced diet. These changes could also result in heightened vulnerability to second hits as has been reported by feeding these KOs a high-fat diet [67]. These insights underline the importance of PEMT in maintaining metabolic health and pave the way for future research into age-related liver dysfunctions.

## Conclusions

Our research provides compelling evidence that PEMT deficiency promotes the spontaneous development of progressive liver disease at a relatively young age, despite being fed a standard diet. The present study underscores the role of PEMT-mediated PC production in preventing liver injury.

## Clinical perspectives

- The enzyme PEMT is a vital enzyme in hepatic phospholipid metabolism, and human polymorphisms leading to its reduced function have been epidemiologically linked to an increased risk of MASLD.
- In the present study, deletion of PEMT in mice was sufficient to spontaneously induce a progressive liver pathology: from steatosis and reduced SAM:SAH ratio to chronic inflammation (MASH), bile acid accumulation, and severe hepatic fibrosis, even on a standard diet.
- These findings establish PEMT deficiency as a primary driver of advanced liver disease, highlighting its potential utility as a critical early risk predictor and a novel therapeutic target for preventing fibrotic progression in MASLD and CLD patients.

## Supplementary Material

Supplementary Figure S1

## Data Availability

Data will be provided upon request.

## Open Access

Open access for this article was enabled by the participation of the University of Nebraska Medical Center in an all-inclusive *Read & Publish* agreement with Portland Press and the Biochemical Society under an agreement with the Big Ten Alliance.

## Abbreviations

α-SMA: smooth muscle actin-α
ALT: alanine aminotransferase
ALD: alcohol-associated liver disease
BHMT: betaine–homocysteine methyltransferase
CD68: cluster of differentiation 68
CLD: chronic liver disease
CYP7A1: cholesterol 7-alpha-monooxygenase
CYP8B1: cytochrome P450 family 8 subfamily B member 1
DAPI: 4′,6-diamidino-2-phenylindole
H&E: hematoxylin and eosin
HNE: hydroxynonenal
HPLC: high-performance liquid chromatography
IL-1β: interleukin-1β
KO: knockout
LAL: lysosomal acid lipase
MASH: metabolic dysfunction-associated steatohepatitis
MASLD: metabolic dysfunction-associated steatotic liver disease
MDA: malondialdehyde
NLRP3: nucleotide-binding domain, leucine-rich repeat, and pyrin domain-containing protein 3
PC: phosphatidylcholine
PE: phosphatidylethanolamine
PEMT: phosphatidylethanolamine N-methyltransferase
ROS: reactive oxygen species
SAH: S-adenosylhomocysteine
SAM: S-adenosylmethionine
TBARS: thiobarbituric acid-reactive substances
TGF-β: transforming growth factor β
TIMP2: tissue inhibitors of metalloproteinases 2
VLDL: very-low-density lipoprotein
WT: wildtype

## References

[1] Lemoinne S. and Friedman S.L. (2019) New and emerging anti-fibrotic therapeutics entering or already in clinical trials in chronic liver diseases. Curr. Opin. Pharmacol. 49, 60–70 10.1016/j.coph.2019.09.006 31590120 31590120

[2] Salarian M., Turaga R.C., Xue S., Nezafati M., Hekmatyar K., Qiao J. et al. (2019) Early detection and staging of chronic liver diseases with a protein MRI contrast agent. Nat. Commun. 10, 4777 10.1038/s41467-019-11984-2 31664017 PMC6820552

[3] Younossi Z.M., Marchesini G., Pinto-Cortez H. and Petta S. (2019) Epidemiology of nonalcoholic fatty liver disease and nonalcoholic steatohepatitis: implications for liver transplantation. Transplantation 103, 22–27 10.1097/TP.0000000000002484 30335697

[4] Marcellin P., Gane E., Buti M., Afdhal N., Sievert W., Jacobson I.M. et al. (2013) Regression of cirrhosis during treatment with tenofovir disoproxil fumarate for chronic hepatitis B: a 5-year open-label follow-up study. Lancet 381, 468–475 10.1016/S0140-6736(12)61425-1 23234725

[5] Watkins S.M., Zhu X. and Zeisel S.H. (2003) Phosphatidylethanolamine-N-methyltransferase activity and dietary choline regulate liver-plasma lipid flux and essential fatty acid metabolism in mice. J. Nutr. 133, 3386–3391 10.1093/jn/133.11.3386 14608048

[6] Reo N.V., Adinehzadeh M. and Foy B.D. (2002) Kinetic analyses of liver phosphatidylcholine and phosphatidylethanolamine biosynthesis using (13)C NMR spectroscopy. Biochim. Biophys. Acta 1580, 171–188 10.1016/S1388-1981(01)00202-5 11880242

[7] Sundler R. and Akesson B. (1975) Regulation of phospholipid biosynthesis in isolated rat hepatocytes. Effect of different substrates. J. Biol. Chem. 250, 3359–3367 10.1016/S0021-9258(19)41523-8 1123345

[8] DeLong C.J., Shen Y.J., Thomas M.J. and Cui Z. (1999) Molecular distinction of phosphatidylcholine synthesis between the CDP–choline pathway and phosphatidylethanolamine methylation pathway. J. Biol. Chem. 274, 29683–29688 10.1074/jbc.274.42.29683 10514439

[9] Vance D.E. (2014) Phospholipid methylation in mammals: from biochemistry to physiological function. Biochim. Biophys. Acta 1838, 1477–1487 10.1016/j.bbamem.2013.10.018 24184426

[10] Vance D.E. (2013) Physiological roles of phosphatidylethanolamine N-methyltransferase. Biochim. Biophys. Acta 1831, 626–632 10.1016/j.bbalip.2012.07.017 22877991

[11] Keogh M.R., Courtney P.D., Kinney A.J. and Dewey R.E. (2009) Functional characterization of phospholipid N-methyltransferases from *Arabidopsis* and soybean. J. Biol. Chem. 284, 15439–15447 10.1074/jbc.M109.005991 19366698 PMC2708841

[12] Gaynor P.M. and Carman G.M. (1990) Phosphatidylethanolamine methyltransferase and phospholipid methyltransferase activities from *Saccharomyces cerevisiae*. Enzymological and kinetic properties. Biochim. Biophys. Acta 1045, 156–163 10.1016/0005-2760(90)90145-N 2198947

[13] Nishimaki-Mogami T., Yao Z. and Fujimori K. (2002) Inhibition of phosphatidylcholine synthesis via the phosphatidylethanolamine methylation pathway impairs incorporation of bulk lipids into VLDL in cultured rat hepatocytes. J. Lipid Res. 43, 1035–1045 10.1194/jlr.M100354-JLR200 12091487

[14] Noga A.A., Zhao Y. and Vance D.E. (2002) An unexpected requirement for phosphatidylethanolamine N-methyltransferase in the secretion of very low density lipoproteins. J. Biol. Chem. 277, 42358–42365 10.1074/jbc.M204542200 12193594

[15] Li J., Xin Y., Li J., Chen H. and Li H. (2023) Phosphatidylethanolamine N-methyltransferase: from functions to diseases. Aging Dis. 14, 879–891 10.14336/AD.2022.1025 37191416 PMC10187709

[16] Kharbanda K.K. (2007) Role of transmethylation reactions in alcoholic liver disease. World J. Gastroenterol. 13, 4947–4954 10.3748/wjg.v13.i37.4947 17854136 PMC4434617

[17] Kharbanda K.K., Todero S.L., Ward B.W., Cannella J.J. and 3rdand Tuma D.J. (2009) Betaine administration corrects ethanol-induced defective VLDL secretion. Mol. Cell. Biochem. 327, 75–78 10.1007/s11010-009-0044-2 19219625

[18] Zhou Q., Zhang F., Kerbl-Knapp J., Korbelius M., Kuentzel K.B., Vujic N. et al. (2022) Phosphatidylethanolamine N-methyltransferase knockout modulates metabolic changes in aging mice. Biomolecules 12, 1270 10.3390/biom12091270 36139111 PMC9496051

[19] Song J., da Costa K.A., Fischer L.M., Kohlmeier M., Kwock L., Wang S. et al. (2005) Polymorphism of the PEMT gene and susceptibility to nonalcoholic fatty liver disease (NAFLD). FASEB J. 19, 1266–1271 10.1096/fj.04-3580com 16051693 PMC1256033

[20] Bale G., Vishnubhotla R.V., Mitnala S., Sharma M., Padaki R.N., Pawar S.C. et al. (2019) Whole-exome sequencing identifies a variant in phosphatidylethanolamine N-methyltransferase gene to be associated with lean-nonalcoholic fatty liver disease. J. Clin. Exp. Hepatol. 9, 561–568 10.1016/j.jceh.2019.02.001 31695245 PMC6823660

[21] Nakatsuka A., Matsuyama M., Yamaguchi S., Katayama A., Eguchi J., Murakami K. et al. (2016) Insufficiency of phosphatidylethanolamine N-methyltransferase is risk for lean non-alcoholic steatohepatitis. Sci. Rep. 6, 21721 10.1038/srep21721 26883167 PMC4756298

[22] Walkey C.J., Donohue L.R., Bronson R., Agellon L.B. and Vance D.E. (1997) Disruption of the murine gene encoding phosphatidylethanolamine N-methyltransferase. Proc. Natl. Acad. Sci. U. S. A. 94, 12880–12885 10.1073/pnas.94.24.12880 9371769 PMC24232

[23] Kharbanda K.K., Mailliard M.E., Baldwin C.R., Beckenhauer H.C., Sorrell M.F. and Tuma D.J. (2007) Betaine attenuates alcoholic steatosis by restoring phosphatidylcholine generation via the phosphatidylethanolamine methyltransferase pathway. J. Hepatol. 46, 314–321 10.1016/j.jhep.2006.08.024 17156888

[24] Kleiner D.E., Brunt E.M., Van Natta M., Behling C., Contos M.J., Cummings O.W. et al. (2005) Design and validation of a histological scoring system for nonalcoholic fatty liver disease. Hepatology 41, 1313–1321 10.1002/hep.20701 15915461

[25] Kharbanda K.K., Todero S.L., Moats J.C., Harris R.M., Osna N.A., Thomes P.G. et al. (2014) Alcohol consumption decreases rat hepatic creatine biosynthesis via altered guanidinoacetate methyltransferase activity. Alcohol Clin. Exp. Res. 38, 6, 648 10.1111/acer.12306 24256608

[26] Folch J., Lees M. and Sloane Stanley G.H. (1957) A simple method for the isolation and purification of total lipides from animal tissues. J. Biol. Chem. 226, 497–509 10.1016/S0021-9258(18)64849-5 13428781

[27] Fuchs C.D., Simbrunner B., Baumgartner M., Campbell C., Reiberger T. and Trauner M. (2025) Bile acid metabolism and signalling in liver disease. J. Hepatol. 82, 134–153 10.1016/j.jhep.2024.09.032 39349254

[28] Ericson L.E. and Harper A.E. (1956) Effect of diet on the betaine-homocysteine transmethylase activity of rat liver I amino acids and proteins. J. Biol. Chem. 219, 49–58 10.1016/S0021-9258(18)65768-0 13295255

[29] Koc H., Mar M.H., Ranasinghe A., Swenberg J.A. and Zeisel S.H. (2002) Quantitation of choline and its metabolites in tissues and foods by liquid chromatography/electrospray ionization–isotope dilution mass spectrometry. Anal. Chem. 74, 4734–4740 10.1021/ac025624x 12349977

[30] Rodrigues Siqueira I., Fochesatto C., da Silva Torres I.L., Dalmaz C. and Alexandre Netto C. (2005) Aging affects oxidative state in hippocampus, hypothalamus and adrenal glands of Wistar rats. Life Sci. 78, 271–278 10.1016/j.lfs.2005.04.044 16112138

[31] Bradford M.M. (1976) A rapid and sensitive method for the quantitation of microgram quantities of protein utilizing the principle of protein-dye binding. Anal. Biochem. 72, 248–254 10.1016/0003-2697(76)90527-3 942051

[32] Mihara M. and Uchiyama M. (1978) Determination of malonaldehyde precursor in tissues by thiobarbituric acid test. Anal. Biochem. 86, 271–278 10.1016/0003-2697(78)90342-1 655387

[33] Osna N.A., Donohue T.M. Jr. and Kharbanda K.K. (2017) Alcoholic liver disease: pathogenesis and current management. Alcohol. Res. 38, 147–161 10.35946/arcr.v38.2.01 28988570 PMC5513682

[34] Osna N.A., White R.L., Donohue T.M. Jr., Beard M.R., Tuma D.J. and Kharbanda K.K. (2010) Impaired methylation as a novel mechanism for proteasome suppression in liver cells. Biochem. Biophys. Res. Commun. 391, 1291–1296 10.1016/j.bbrc.2009.12.074 20026058 PMC2812660

[35] Osna N.A., White R.L., Krutik V.M., Wang T., Weinman S.A. and Donohue T.M. Jr. (2008) Proteasome activation by hepatitis C core protein is reversed by ethanol-induced oxidative stress. Gastroenterology 134, 2144–2152 10.1053/j.gastro.2008.02.063 18549882 PMC2517112

[36] Barrett A.J. and Kirschke H. (1981) Cathepsin B, cathepsin H, and cathepsin L. Methods Enzymol. 80 Pt C, 535–561 10.1016/S0076-6879(81)80043-2 7043200

[37] Yan C., Lian X., Li Y., Dai Y., White A., Qin Y. et al. (2006) Macrophage-specific expression of human lysosomal acid lipase corrects inflammation and pathogenic phenotypes in *lal*^-/-^ mice. Am. J. Pathol. 169, 916–926 10.2353/ajpath.2006.051327 16936266 PMC1698822

[38] Perumal S.K., Day L.Z., Arumugam M.K., Chava S., Kumar V., Osna N.A. et al. (2024) Lipid droplet-associated proteins in alcohol-associated fatty liver disease: a proteomic approach. Alcohol. Clin. Exp. Res. (Hoboken) 48, 2010–2021 10.1111/acer.15446 39414381 PMC11778054

[39] Kharbanda K.K., Rogers D.D. 2nd, Mailliard M.E., Siford G.L., Barak A.J., Beckenhauer H.C. et al. (2005) Role of elevated *S*-adenosylhomocysteine in rat hepatocyte apoptosis: protection by betaine. Biochem. Pharmacol. 70, 1883–1890 10.1016/j.bcp.2005.09.021 16253211

[40] Arumugam M.K., Chava S., Perumal S.K., Paal M.C., Rasineni K., Ganesan M. et al. (2022) Acute ethanol-induced liver injury is prevented by betaine administration. Front. Physiol. 13, 940148 10.3389/fphys.2022.940148 36267591 PMC9577233

[41] van der Veen J.N., Lingrell S., Gao X., Takawale A., Kassiri Z., Vance D.E. et al. (2017) Fenofibrate, but not ezetimibe, prevents fatty liver disease in mice lacking phosphatidylethanolamine N-methyltransferase. J. Lipid Res. 58, 656–667 10.1194/jlr.M070631 28159867 PMC5392742

[42] Martinez-Una M., Varela-Rey M., Mestre D., Fernandez-Ares L., Fresnedo O., Fernandez-Ramos D. et al. (2015) *S*-Adenosylmethionine increases circulating very-low density lipoprotein clearance in non-alcoholic fatty liver disease. J. Hepatol. 62, 673–681 10.1016/j.jhep.2014.10.019 25457203 PMC4336596

[43] Roehlen N., Crouchet E. and Baumert T.F. (2020) Liver fibrosis: mechanistic concepts and therapeutic perspectives. Cells 9, 875 10.3390/cells9040875 32260126 PMC7226751

[44] Musso G., Saba F., Cassader M. and Gambino R. (2023) Lipidomics in pathogenesis, progression and treatment of nonalcoholic steatohepatitis (NASH): Recent advances. Prog. Lipid Res. 91, 101238 10.1016/j.plipres.2023.101238 37244504

[45] Barrios J.M. and Lichtenberger L.M. (2000) Role of biliary phosphatidylcholine in bile acid protection and NSAID injury of the ileal mucosa in rats. Gastroenterology 118, 1179–1186 10.1016/S0016-5085(00)70371-4 10833493

[46] Kasbo J., Tuchweber B., Perwaiz S., Bouchard G., Lafont H., Domingo N. et al. (2003) Phosphatidylcholine-enriched diet prevents gallstone formation in mice susceptible to cholelithiasis. J. Lipid Res. 44, 2297–2303 10.1194/jlr.M300180-JLR200 12837851

[47] Li M., Cai S.Y. and Boyer J.L. (2017) Mechanisms of bile acid mediated inflammation in the liver. Mol. Aspects Med. 56, 45–53 10.1016/j.mam.2017.06.001 28606651 PMC5662014

[48] Morita S.Y., Ikeda Y., Tsuji T. and Terada T. (2019) Molecular mechanisms for protection of hepatocytes against bile salt cytotoxicity. Chem. Pharm. Bull. (Tokyo) 67, 333–340 10.1248/cpb.c18-01029 30930437

[49] Stead L.M., Brosnan J.T., Brosnan M.E., Vance D.E. and Jacobs R.L. (2006) Is it time to reevaluate methyl balance in humans? Am. J. Clin. Nutr. 83, 5–10 10.1093/ajcn/83.1.5 16400042

[50] Kharbanda K.K. (2013) Methionine metabolic pathway in alcoholic liver injury. Curr. Opin. Clin. Nutr. Metab. Care 16, 89–95 10.1097/MCO.0b013e32835a892a 23232418

[51] Kharbanda K.K. (2009) Alcoholic liver disease and methionine metabolism. Sem Liver Dis. 29, 155–165 10.1055/s-0029-1214371 19387915

[52] Barak A.J., Baker H. and Tuma D.J. (1981) Influence of ethanol on *in-vivo* levels of hepatic methylators betaine and N5-methyltetrahydrofolate in the rat. IRCS Med. Sci. 9, 527–528

[53] Nicolaou A., Kenyon S.H., Gibbons J.M., Ast T. and Gibbons W.A. (1996) *In vitro* inactivation of mammalian methionine synthase by nitric oxide. Eur. J. Clin. Invest. 26, 167–170 10.1046/j.1365-2362.1996.122254.x 8904527

[54] Nicolaou A., Waterfield C.J., Kenyon S.H. and Gibbons W.A. (1997) The inactivation of methionine synthase in isolated rat hepatocytes by sodium nitroprusside. Eur. J. Biochem. 244, 876–882 10.1111/j.1432-1033.1997.00876.x 9108260

[55] Barak A.J., Beckenhauer H.C. and Tuma D.J. (2002) Methionine synthase: a possible prime site of the ethanolic lesion in liver. Alcohol 26, 65–67 10.1016/S0741-8329(01)00201-4 12007580

[56] Osna N.A., White R.L., Donohue T.M. Jr., Tuma D.J. and Kharbanda K.K. (2009) Impaired methylation reduces proteasome activity in liver cells. Alcohol. Clin. Exp. Res. 33, 229A, Abstract #873

[57] Ganesan M., Feng D., Barton R.W., Thomes P.G., McVicker B.L., Tuma D.J. et al. (2016) Creatine supplementation does not prevent the development of alcoholic steatosis. Alcohol. Clin. Exp. Res. 40, 2312–2319 10.1111/acer.13214 27581622

[58] Yadati T., Houben T., Bitorina A. and Shiri-Sverdlov R. (2020) The ins and outs of cathepsins: physiological function and role in disease management. Cells 9, 1679 10.3390/cells9071679 32668602 PMC7407943

[59] Donohue T.M. Jr., Osna N.A., Kharbanda K.K. and Thomes P.G. (2019) Lysosomal and proteasome dysfunction in alcohol-induced liver injury. Liver Res. 3, 191–205 10.1016/j.livres.2019.11.001

[60] Kraus S., Bunsen T., Schuster S., Cichon M.A., Tacke M., Reinheckel T. et al. (2011) Cellular senescence induced by cathepsin X downregulation. Eur. J. Cell Biol. 90, 678–686 10.1016/j.ejcb.2011.03.008 21616554

[61] Bellanti F., Villani R., Facciorusso A., Vendemiale G. and Serviddio G. (2017) Lipid oxidation products in the pathogenesis of non-alcoholic steatohepatitis. Free Radic. Biol. Med. 111, 173–185 10.1016/j.freeradbiomed.2017.01.023 28109892

[62] Castro J.P., Jung T., Grune T. and Siems W. (2017) 4-Hydroxynonenal (HNE) modified proteins in metabolic diseases. Free Radic. Biol. Med. 111, 309–315 10.1016/j.freeradbiomed.2016.10.497 27815191

[63] Luangmonkong T., Suriguga S., Mutsaers H.A.M., Groothuis G.M.M., Olinga P. and Boersema M. (2018) Targeting oxidative stress for the treatment of liver fibrosis. Rev. Physiol. Biochem. Pharmacol. 175, 71–102 10.1007/112_2018_10 29728869

[64] Sanchez-Valle V., Chavez-Tapia N.C., Uribe M. and Mendez-Sanchez N. (2012) Role of oxidative stress and molecular changes in liver fibrosis: a review. Curr. Med. Chem. 19, 4850–4860 10.2174/092986712803341520 22709007

[65] Heid M.E., Keyel P.A., Kamga C., Shiva S., Watkins S.C. and Salter R.D. (2013) Mitochondrial reactive oxygen species induces NLRP3-dependent lysosomal damage and inflammasome activation. J. Immunol. 191, 5230–5238 10.4049/jimmunol.1301490 24089192 PMC3833073

[66] Dadmanesh M., Ranjbar M.M. and Ghorban K. (2019) Inflammasomes and their roles in the pathogenesis of viral hepatitis and their related complications: an updated systematic review. Immunol. Lett. 208, 11–18 10.1016/j.imlet.2019.03.001 30831142 PMC7112799

[67] Jacobs R.L., Zhao Y., Koonen D.P., Sletten T., Su B., Lingrell S. et al. (2010) Impaired *de novo* choline synthesis explains why phosphatidylethanolamine N-methyltransferase-deficient mice are protected from diet-induced obesity. J. Biol. Chem. 285, 22403–22413 10.1074/jbc.M110.108514 20452975 PMC2903412

