# Supplementary material for: Deletion of phosphatidylethanolamine methyltransferase promotes the spontaneous development of hepatic steatosis, inflammation, and fibrosis in young mice
Source: Clin Sci (Lond). 2026 Jun 29;140(7):1535–49. doi: 10.1042/CS20250454 (PMC13314529; doi:10.1042/CS20250454)
Supplement: Supplementary Figure S1 [file CS-2025-0454_supp.pdf]

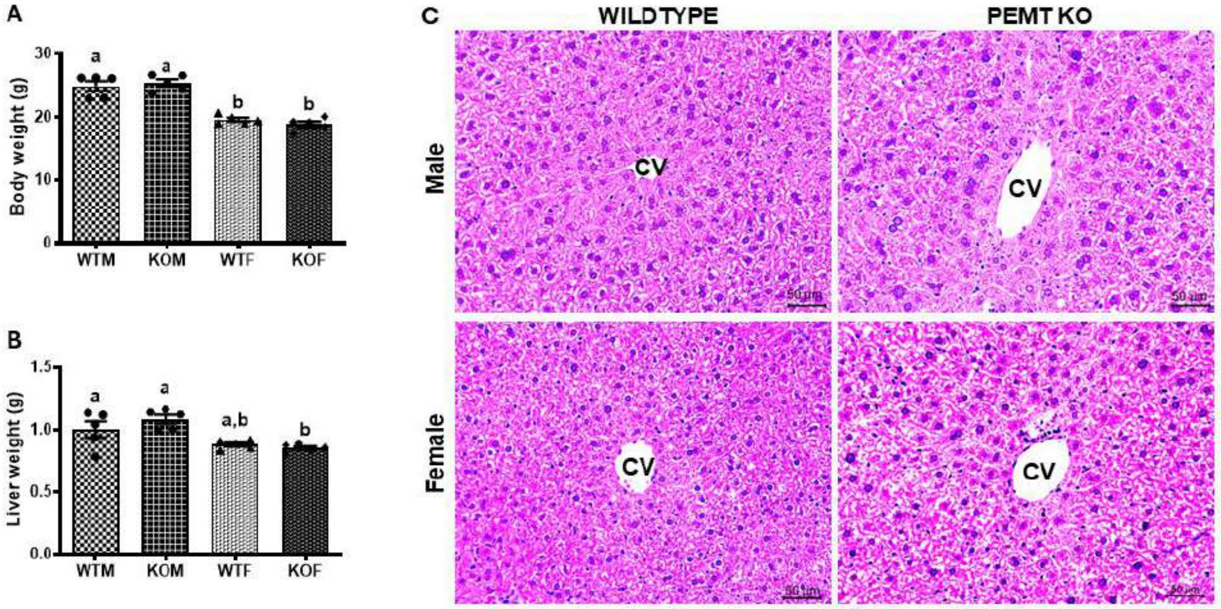


**Figure S1. Body weight, liver weight and hepatic histology of 2-month-old PEMT-deficient mice**. A) Body weight, B) liver weight, C) Representative images of hematoxylin and eosin-stained liver sections (Scale bar: 50-µm) from 2-month-old male and female PEMT KO mice (KOM and KOF, respectively) compared with their respective age- and sex-matched wildtype controls (WTM and WTF). Data are presented as the mean ± SEM (n=4-6); values not sharing a common letter significantly differ from each other at $p \leq 0.05$.
